# Relationship Between Entrepreneurship Education Curriculum and Agricultural Students’ Satisfaction in China

**DOI:** 10.3389/fpsyg.2022.884565

**Published:** 2022-06-09

**Authors:** Yangjie Huang, Yajing Bu, Lanying Liu, Da Xu, Zengliu Xu, Guojing Zhao

**Affiliations:** ^1^School of Education, Hangzhou Normal University, Hangzhou, China; ^2^Institute of China Innovation and Entrepreneurship Education, Wenzhou Medical University, Wenzhou, China; ^3^College of Landscape Architecture, Zhejiang A&F University, Hangzhou, China; ^4^Student Affairs Office, China Academy of Art, Hangzhou, China

**Keywords:** curriculum, entrepreneurship practice, satisfaction, entrepreneurship education, agricultural students

## Abstract

Developing agriculture is an important way to get rid of poverty and boost economic development. Entrepreneurship, especially entrepreneurship education, is considered to be an important contributor to the realization of the above objectives. Entrepreneurship education has received more and more attention. Improving the entrepreneurial willingness and skills of agricultural students is of great significance to the economic and social development of developing countries. In order to explore the relationship between entrepreneurship education curricula and satisfaction, especially the agricultural students, we conducted a questionnaire survey in 35 universities across the country between 2018 and 2019 to evaluate the entrepreneurship education of agricultural students in Chinese universities. And 1223 valid questionnaires with 7 interviews were obtained. Furthermore, we used the structural equation model to empirically analyze the questionnaire data and found that entrepreneurship practice plays a part in the mediating effect between entrepreneurship curriculum and satisfaction with entrepreneurship education. This paper expands literature on entrepreneurship education and has certain reference significance for training a large number of agricultural entrepreneurial talents the practice of entrepreneurship education in other developing countries.

## Introduction

Since the 21st century, with the development of digital economy, innovative, and entrepreneurial talents are the first strategic resource, which has basically formed an international consensus (Löfsten et al., 2020). At present, developed countries such as Europe and the United States attach great importance to the role of innovation and entrepreneurship in promoting national economic development (Youssef et al., 2018). Similarly, entrepreneurship is also a basic problem in the economies of developing countries. The economic development or decline of a country mainly depends on the existence and role of entrepreneur groups (Stoica et al., 2020). In particular, universities are regarded as the main actors and triggers of technological change and innovation, as well as important tools of regional economic and social development and knowledge production (Genc et al., 2020). Therefore, entrepreneurship education in universities is of great significance to the sustainable development of developing countries. Entrepreneurship curriculum and entrepreneurship practice are the main channels for the implementation of entrepreneurship education. To a certain extent, they can affect students’ satisfaction with entrepreneurship education and reflect the quality of entrepreneurship education.

However, at present, scholars mainly discuss entrepreneurship education dominated by engineering students such as electrical engineering (Ismail et al., 2018), and there is little literature on entrepreneurship education for agricultural students. Although mainstream entrepreneurship research previously ignored agriculture and its related industries, this situation has changed in recent years (Afreh et al., 2019). In the face of changing political, economic, social, and natural environmental factors, we need to re-examine the agricultural sector with new vitality in order to promote the sustainable development of other sectors and human survival (Lin, 2022). Obviously, it is expected that the development of agricultural entrepreneurship will play a key role in poverty alleviation and wealth creation (Bateman et al., 2019). As a large agricultural country, Three Rural Problems (Problems about Agriculture, Rural areas, and Peasantry) have always been major issues related to the national economy and the people’s livelihood in China. At present, China’s Rural Revitalization Strategy urgently needs talents with innovative spirit and entrepreneurial ability for intellectual support (Long et al., 2016). Colleges and universities are important positions for disseminating agricultural scientific knowledge and cultivating agricultural scientific and technological talents. What is the specific curriculum and practice mode of colleges and universities in the cultivation of innovative and entrepreneurial talents of specific agricultural majors; How effective the implementation of entrepreneurship education is and how satisfied the students are; How much impact does curriculum education have on College Students’ entrepreneurial practice? The clarification and answers to these questions are of great significance to improve agricultural innovation and entrepreneurship education.

Scholars have done a lot of research on entrepreneurship education, but most of them belong to theoretical discussion and policy suggestions, and there is little empirical analysis on entrepreneurship education, especially the satisfaction of entrepreneurship education. Based on this, this paper takes 1223 agricultural students from 35 universities in 20 provinces and cities in China as samples, and uses structural equation model to analyze the relationships between entrepreneurship curricula, entrepreneurship practice, and entrepreneurship education satisfaction. On this basis, this paper puts forward some suggestions on entrepreneurship education for agricultural college students, in order to establish agricultural entrepreneurship education close to the real needs of college students, fully cultivate college students’ entrepreneurial ability and entrepreneurial literacy, and provide targeted opinions for effective entrepreneurship education.

The rest of this paper is arranged as follows: the second chapter introduces the relevant literature of agricultural entrepreneurship education and seven interview cases, and makes research hypotheses; the third chapter introduces the data and empirical strategies; the fourth chapter reports the research results; the fifth and sixth chapters are the summary and discussion.

## Literature Review and Hypotheses

### Entrepreneurship Education

As an educational form and social practice, entrepreneurship education changes people and has an impact on students (Liu et al., 2020). The development of entrepreneurship education fills the gap of higher education, and it develops students’ skills, abilities, and knowledge base (Turner and Gianiodis, 2018). This is in line with Fayolle et al.’s (2014) view that entrepreneurship education is an effective means to internalize various experiences, knowledge, values, and norms to students.

American universities have established clear goals for entrepreneurship education, which aims to cultivate the entrepreneurial spirit of each student, serve students’ academic and career development, improve economic and social development, and create social vitality for the social mission. Japan’s entrepreneurship education aims to help students cultivate a broader sense of entrepreneurship through multidisciplinary combinations such as management and political science or sociology and engineering art. Such integration can promote practice research and fieldwork (Nakao and Nishide, 2020). In contrast, in developing countries such as China and Iran, most colleges and universities adopt a single entrepreneurship education model, which is separated from professional education, and pay insufficient attention to students’ practical skills (Yaghoubi, 2010). Moreover, some scholars believe that the entrepreneurship practice for college students in China has some shortcomings, such as insufficient coupling of ecological elements in entrepreneurship practice, disconnection between in-class teaching and extra-curricular activities, lack of practical resources and large gap in teaching requirements for diverse talents (Yinjun et al., 2018). Generally speaking, agricultural colleges and universities lack the practicality and systematicness of agricultural entrepreneurship education in both scientific research and teaching.

### Agricultural Entrepreneurship

Agricultural entrepreneurship is defined as a single or collective effort by an individual or team to develop resources for production of agricultural products and market distribution of relevant useful agricultural products, services or commodities to meet market demand (Spais, 2010; Pliakoura et al., 2020). According to statistics, the number of graduates in China in 2022 reached 10.76 million, and the employment situation is quite grim. Most agricultural graduates come from rural areas. Due to the low salary and hard work, the counterpart employment rate is very low. The same is true in Iran, especially in the agricultural sector (Shiri et al., 2017). Especially affected by the epidemic, the unemployment rate of young men and women around the world remains high (Fegert et al., 2020). Entrepreneurship has been declared by many countries as one of the ways to solve the crisis, and it is also widely regarded as a means of economic development and employment growth (Liu et al., 2020). Obviously, entrepreneurship has become one (or perhaps the most important) aspect of agricultural development in the last decade and will become increasingly important in the near future. Market developments, agricultural policy and society as a whole have emphasized the need for a higher level of entrepreneurship (Beldman et al., 2014). Due to the reciprocal relationship between education and social development and entrepreneurship, agricultural education will continue to play an important role in achieving sustainable agriculture. Therefore, this paper mainly focuses on the entrepreneurship education of agricultural students.

### Entrepreneurship Education Curriculum

University entrepreneurship education is widely popular, and many agribusiness educational institutes also have incorporated the concepts of entrepreneurship into their curriculum (Higgins et al., 2018). Curriculum is the core element of school education, and the quality of curriculum directly determines the quality of talent training (Wang et al., 2022). Only through curriculum education can we realize the strong intellectual support of entrepreneurial talents in the process of rural economic development. At the same time, this is also an important aspect of students’ quality training, which is conducive to improving their comprehensive quality and ability and achieving the ultimate goal of talent training in colleges and universities (Wang, 2019). Entrepreneurship curriculum is the key carrier, core channel and main form of entrepreneurship education in colleges and universities. It has unique relevance as the “observation point” of entrepreneurship education (Hasan et al., 2017).

### Entrepreneurship Practice

As an important part of entrepreneurship education, entrepreneurship practice is an effective extension and enrichment of entrepreneurship education classroom teaching in universities (Wang, 2020a). Entrepreneurship practice is a bridge between theoretical knowledge of entrepreneurship and practical entrepreneurship. Sansone et al. (2021) even further classified the forms of entrepreneurship education and pointed out that practical entrepreneurship education has a greater impact on students than theoretical entrepreneurship education. Scholars Scott et al. (2016) analyzed the effectiveness of experiential learning and emphasized that experiential methods improve students’ ability to identify and take advantage of business opportunities. Song and Yang (2016) found through investigation that entrepreneurship practice with discipline competition as the core has a significant impact on innovation and entrepreneurship education. In a word, entrepreneurship practice is an effective form of entrepreneurship education, which helps to encourage individuals to start their own businesses and stimulate the entrepreneurial spirit and innovation gene of the nation (Yan et al., 2018).

### Satisfaction With Entrepreneurship Education

Student satisfaction is one of the most basic variables to evaluate the quality of educational projects, because it is considered to be an important predictor of the quality of academic experience (Song and Meier, 2022). This concept originates from the concept of customer satisfaction, and the satisfaction of entrepreneurship education studied in this paper refers to a subjective evaluation of college students’ satisfaction with entrepreneurship education (Huang et al., 2020b). Although the research on the evaluation of entrepreneurship education has achieved some results, the evaluation often pays attention to the benefit evaluation indicators, takes students as the objective object of investigation, and often ignores students’ subjective feelings and needs. Students’ satisfaction with entrepreneurship education is an important indicator to measure the quality of entrepreneurship education provided by colleges and universities (Jena, 2020); Byun et al. (2018) found in the survey of students’ satisfaction with entrepreneurship education that students have the highest demand for various curriculum structures and professional lecturers with rich practical experience. As a large agricultural country, Chinese agricultural students will be important talents for building an innovative country in the future. Their satisfaction with innovation and entrepreneurship education will be an important entry point for improving and perfecting innovation and entrepreneurship education.

### Analysis of Seven Interview Cases

Interview method is a face-to-face social interaction process, and semi-structured interview was adopted in this paper. This method is helpful to obtain detailed data, but it requires a lot of manpower and material resources, and the sample size is relatively small. The combination of interview case study and data survey can help us solve the above research problems more comprehensively (Sawang and Unsworth, 2011). We interviewed teachers and students from agricultural universities in various regions. The content of the teacher interview mainly includes the current situation of entrepreneurship education, the construction of entrepreneurship education evaluation system, and the training of entrepreneurship teachers. The content of the student interview mainly includes what they have gained from the entrepreneurship education, and what comments and suggestions they have made for the entrepreneurship education in their universities. From the entrepreneurship competition winning students, outstanding students, and engaged in self-employment graduates selected as the representative interview objects. From the teachers who responsible for entrepreneurship management, entrepreneurship curriculum teachers and entrepreneurship competition instructors selected as the representative of the teachers as interview objects (see Table 1). It is worth noting that the case study provided an important basis for our questionnaire design and research conclusion.

**TABLE 1:** Interviewee list (Details).

No	Name	Enrollment year	Major	No	Name	Professional qualifications	Teaching age
1	Student A	2014	Geographic information science	5	Teacher a	Associate professor	10 years
2	Student B	2018	Botanical garden specialty	6	Teacher b	Lectureship	6 years
3	Student C	2016	Land resources management	7	Teacher c	Teaching assistant	3 years
4	Student D	2015	Land resources management				

In the context of developing countries, our goal is to provide examples from a dual perspective through interviews with teachers and students. First, we adopted a case study approach and ultimately identified seven candidates. Secondly, in the process of data analysis, the reliability and validity of the research are ensured by making interview plans, writing memos and group discussions, etc. Finally, through thematic analysis (Yunis et al., 2018), in-depth reading and rereading interview transcripts to explain their feelings of entrepreneurship education (see Table 2).

**TABLE 2:** Themes explained through interview data.

Themes	Interpretation of raw data through participant interviews
**Students’ knowledge, skills, and quality**	The basic skills of employment, job search, and application are also made substantial improvement. (Student A)
	First, we have learned the knowledge of entrepreneurship, the second is the ability of the venture plan, the commercial powerpoint production, the basic software application ability, and the language expression and the response. (Student C)
	Innovation entrepreneurship is based on specialized curriculum, which has improved my professional skills in practice. (Student D)
**The increase of students’ willingness to start a business**	Theory study can better guide the development of entrepreneurial practice, entrepreneurial awareness, and willingness will naturally increase. (Student A)
	Learn the necessary conditions, relevant policies, and legal knowledge to avoid detours in the process of starting a business and reduce unnecessary losses. (Student B)
	Through study and practice, I have a clearer understanding of the systematic process of entrepreneurship, a more rational understanding of entrepreneurship, and a greater passion for entrepreneurship. (Student D)
**Teaching evaluation of entrepreneurship education**	Entrepreneurship communication is particularly prominent. For example, through alumni returning to school to share entrepreneurial experience, the school conveys cutting-edge entrepreneurial spirit, which plays a far more important role than textbook design, teaching, and assessment in class. However, the school entrepreneurship education guidance teachers slightly weak. (Student A)
	The teacher guides each student to make a business plan one by one according to their different characteristics. (Student B)
	In the innovation and entrepreneurship competition, almost every project will have a special teacher to provide all-round guidance, to provide a strong teacher guarantee for students, but the shortage is the lack of off-campus practice projects. (Student C)
	What helped me the most was the teaching mechanism of entrepreneurship education in university, but it was deficient in teaching materials involved. (Student D)
	Our university attaches great importance to innovation and entrepreneurship education, and carries out innovation and entrepreneurship education curriculum in combination with the characteristics of agricultural majors. By inviting industry experts, employers, and entrepreneurial graduates to jointly formulate the curriculum system and practice system, we form a curriculum system that integrates “basic course-core course-practical course.” (Teacher a)
**Evaluation of entrepreneurship education service support**	Entrepreneurship tutor, entrepreneurship base, there are also some alumni entrepreneurship scholarships. (Student A)
	Freshmen can apply for retaining the admission qualification to carry out innovation and entrepreneurship practice, and can also apply for suspension of schooling to carry out entrepreneurship after enrollment. Students can study in a variety of ways, including applying for cross-school minor or taking curriculum, and students’ credit accumulation and recognition system are clearly defined for participating in the open online curriculum recognized by the school. (Student B)
**Practice evaluation of entrepreneurship education**	The entrepreneurial activities on campus and off campus, as well as the risks and pressures faced by entrepreneurs, are never at the same level. Therefore, the entrepreneurial practice that can truly learn something is based on the campus and expanded off campus. (Student C)
	Through theoretical study and entrepreneurial practice, I have a better understanding of the market environment and entrepreneurial prospects. For example, our teacher analyzed the stimulating and guiding effect that the hot policies like “beautiful rural construction” and “targeted poverty alleviation” might have on the market in the next step. Based on this, I organized and participated in several environmental protection agricultural science and technology projects in university, and won the approval of the judges in many innovation and entrepreneurship competitions. (Student D)
**Evaluation of entrepreneurship education**	Students’ evaluation of university entrepreneurship education should be based on the experience of participating in entrepreneurial activities and the degree of improvement of practical ability. (Student D)
	It should be evaluated from the five aspects of the idea, spirit, knowledge, consciousness, and ability of innovation and entrepreneurship. (Teacher a)
	Short-term evaluation needs to be combined with long-term evaluation. (Teacher b)
	The evaluation should be diversified and comprehensive, not only looking at one or two indicators, but also running through the whole university education, and even the survey of graduates should be timely interviewed and tracked. (Teacher c)

Through cases study, this study found that Entrepreneurship education of agricultural students should be comprehensive and practical. It should not only stay in the classroom. At the same time, entrepreneurship education should be precisely segmented. Different industries and different entrepreneurial groups should be professionally guided by teachers and entrepreneurs of different specialties (Student A). Therefore, in the process of instructing students, teachers should not only have solid basic knowledge of entrepreneurship, but also have experience of entrepreneurship, be able to guide students to carry out various entrepreneurial practice simulation activities, help students solve problems arising from entrepreneurial practice, and improve students’ entrepreneurial awareness and ability (Teacher b). Teachers should adopt new teaching mode and encourage teachers to deeply integrate professional curriculum and entrepreneurship education (Huang et al., 2020a).

At the same time, entrepreneurial practice is more meaningful. Successful entrepreneurship education can not only impart student’s entrepreneurship knowledge, but more importantly, it should provide students with sufficient practical opportunities, encourage students to actively develop entrepreneurial thinking, so that students have a more comprehensive understanding of entrepreneurship, with higher entrepreneurial literacy. Entrepreneurship education gives students many opportunities to personally participate in entrepreneurship practice, and breaks the barriers of limited entrepreneurial experience for young entrepreneurs, which is no longer just an “armchair strategy” (Student D).

### Hypothesized

As mentioned above, scholars have emphasized that entrepreneurship education curriculum, as an important carrier of Entrepreneurship Talent Training, plays an irreplaceable role in the teaching of entrepreneurial knowledge and the cultivation of entrepreneurial skills. Through empirical research, Azila-Gbettor and Harrison (2013) found that due to the lack of applicability of entrepreneurship courses and the inability to meet the needs of students, graduates are unable to make use of their learned knowledge when obtaining off campus entrepreneurship resources, resulting in low satisfaction with entrepreneurship education in the university. Badariah et al. (2015) used survey techniques to evaluate the effectiveness of entrepreneurship education programs in Malaysian public universities. The results showed that the entrepreneurship curriculum was very effective in improving students’ entrepreneurial skills, and universities could improve students’ entrepreneurial skills by setting up entrepreneurship curricula. So well-designed curricula such as experiential curricula and entrepreneurial activities can increase the benefits of entrepreneurship, improve students’ satisfaction with the entrepreneurship education and increase their willingness to participate in enterprises (Yu and Wang, 2019). Therefore, this paper proposed the following research hypotheses:

**Hypothesis 1**: Entrepreneurship Education curriculum have a positive impact on students’ satisfaction with entrepreneurship education.

Entrepreneurship curricula also have a positive impact on entrepreneurial ability and intention, helping students learn entrepreneurial ability and increase their willingness to become future entrepreneurs (Wang and Huang, 2020). Because the content of entrepreneurship education curriculum is selected and designed based on the talent training objectives of entrepreneurship education. The curriculum system with reasonable structure, rich content, diverse forms, and integration of theory and practice is directly related to the training effect of entrepreneurship education (Mei and Symaco, 2022); Hosseini and Pouratashi (2011) used a random sampling method and selected two experimental groups from a single university, that is, participants of an entrepreneurship curriculum and those of a non-entrepreneurship curriculum. The control group was chosen from another university to evaluate the effect of entrepreneurship curricula on improving the entrepreneurial ability of agricultural students. The results showed that there is a significant difference in the entrepreneurial ability of agricultural students who have attended entrepreneurship curricula and those who have not. So now entrepreneurship education curriculum pays more and more attention to experiential learning, which transforms knowledge into a kind of skill through entrepreneurial practice and has a positive impact on entrepreneurial practice. Therefore, this paper proposed the following research hypotheses:

**Hypothesis 2**: Entrepreneurship Education curriculum have a positive impact on students’ entrepreneurship practices.

As mentioned above, entrepreneurial practice based on experiential learning such as entrepreneurial competition can effectively attract students and significantly improve students’ entrepreneurial quality. Some scholars have pointed out that to improve students’ satisfaction with entrepreneurship education, schools attach importance to entrepreneurship activities and encourage students to conduct such activities and increase their learning experience. Higgins et al. (2018) emphasized the importance of universities enriching students’ experience by utilizing entrepreneurial activities through, for example, corporate partnerships. Entrepreneurship education exposes students to successful business plans or positive interactions with successful practitioners. This pedagogy element provides coping strategies, helps maintain motivation and interest, leads to higher expectations of success, and increases entrepreneurial self-efficacy (Boldureanu et al., 2020); Heinrichs (2016) designed the course content with entrepreneurship cases, which made up for the deficiency of traditional entrepreneurship courses that only focused on the accumulation of theoretical knowledge and ignored practical experience. He effectively improved the satisfaction of entrepreneurship education by sharing successful entrepreneurship cases. Therefore, this paper proposes that entrepreneurship practical curriculum and practical activities have a positive impact on entrepreneurship education satisfaction.

**Hypothesis 3**: Entrepreneurship practice has a positive impact on students’ satisfaction with entrepreneurship education.

**FIGURE 1:**
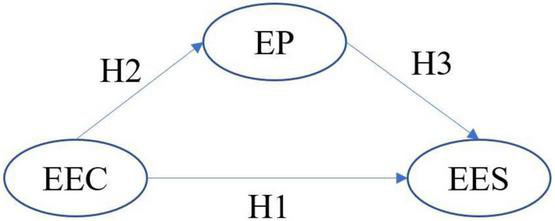
Research framework. EEC, entrepreneurship education curriculum; EP, entrepreneurship practice; EES, entrepreneurship education satisfaction.

Based on this, we propose a hypothetical model (see Figure 1).

## Materials and Methods

### Measurement

This study explored the relationship between entrepreneurship education curriculum, entrepreneurship practice and college students’ satisfaction with entrepreneurship education through quantitative research. We used stratified random sampling in all provinces of China and conducted online questionnaires from 2018 to 2019. Stratified random sampling is a extensively used sampling technique for approximate query processing (Nguyen et al., 2021). We divided agricultural colleges and universities into three levels according to the type of universities in China, namely, “double first-class” universities, ordinary universities and higher vocational colleges, and assigned stratified samples in proportion (3:9:8) to ensure that each level is adequately represented in the overall sample (see Table 3). The questionnaire covered students’ basic information, their cognitive attitude toward entrepreneurship, implementation process and results of entrepreneurship education, and the final evaluation of their satisfaction with entrepreneurship education. In order to fully grasp the real situation of entrepreneurship education of agricultural students in China, the survey was conducted anonymously, and all the data were only used for academic research.

**TABLE 3:** Descriptive statistics (*N* = 1233).

Demographics	Baseline characteristic	Dimension	
		*n*	%
Gender	Female	680	55.60
	Male	543	44.40
Registered permanent residence	Urban	424	34.70
	Rural	799	65.30
School type	”Double first-class” universities	182	14.90
	Ordinary colleges and universities	545	44.60
	Higher vocational college	496	40.50
Student status	Undergraduate	632	51.68
	Junior college student	460	37.61
	Graduate	131	10.71
Entrepreneurial practice	Yes	279	22.80
	No	944	77.20
Entrepreneurial experience of family members	Yes	254	20.80
	No	969	79.20
Intended destination of graduation	Obtain employment	464	37.90
	Entrance for further study	532	43.50
	Start-up business	208	17.00
	Other	19	1.60

### Participants and Procedure

The participants were current college students or graduates who had attended the university in the past 5 years. Finally, we received a valid sample of 1223 agricultural students from 35 universities in 20 provinces and municipalities of China (see Table 3). Of these, 17 universities are in the eastern region, 8 are in the central region, and 10 are in the western region. The study used SPSS 22 analysis software for statistical analysis of samples. The student’s measurement scale used in this study was based on domestic and foreign literature, along with the semi-structured interview analysis of several experts and scholars in entrepreneurship education. The questionnaire was reviewed and revised with experts in the research field. After the initial draft of the questionnaire was designed, it was modified by the assistance of teachers or managers in the field. After several rounds of modification by team members, it was tested in a small scale. Finally, a large-scale survey and research were conducted. In order to fully grasp the real situation of entrepreneurship education of agricultural students in China, the survey was conducted anonymously, and all the data were only used for academic research.

### Data Collection Tools

This questionnaire focuses on the contents of entrepreneurship education curriculum, entrepreneurship practice, and college students’ satisfaction with entrepreneurship education on five-point Likert scale (1 = strongly disagree; 2 = disagree; 3 = average; 4 = agree; 5 = strongly agree), with a total of 3 dimensions and 17 items. The dimensions of students’ entrepreneurship education in universities are shown in Table 4. IBM SPSS and Amos software were used to test the reliability of the scale (see Table 5).

**TABLE 4:** The dimensions of students’ entrepreneurship education in universities.

The dimensions	Mean value	Standard deviation	Literature support of indicators
**Entrepreneurship education curriculum (EEC)**			Hosseini and Pouratashi, 2011 Wiley and Berry, 2015
There are various types of entrepreneurship education curriculum (EEC1)	3.640	1.047	
Entrepreneurship curriculum content closely combined with professional knowledge (EEC2)	3.594	1.074	
Entrepreneurship curriculum content closely combined with cutting-edge trends closely (EEC3)	3.708	1.023	
Teachers teach in a variety of ways (EEC4)	3.726	1.021	
Teachers have entrepreneurial experience (EEC5)	3.681	1.023	
Teachers have rich experience in entrepreneurship education and teaching (EEC6)	3.790	1.014	
**Entrepreneurship practice (EP)**			Pittaway and Edwards, 2012 Wang, 2020a
There are teachers in and out of campus to guide entrepreneurship practice (EP1)	3.851	0.979	
Entrepreneurial practice is supported by special venture funds (EP2)	3.740	1.022	
The university provides integrated entrepreneurial practice services (EP3)	3.733	1.003	
There are independent student entrepreneurship practice parks (EP4)	3.805	0.996	
There are special off-campus practice bases (EP5)	3.771	0.995	
Entrepreneurial practice programs are highly integrated with professional learning (EP6)	3.745	1.002	
**Satisfaction with entrepreneurship education (EES)**			James, 2014 Huang et al., 2020b Chen et al., 2021
Enrich entrepreneurial knowledge (EES1)	3.913	0.947	
Cultivate the innovative spirit (EES2)	3.923	0.958	
Improve entrepreneurial skills (EES3)	3.936	0.957	
Inspire entrepreneurial willingness (EES4)	3.944	0.966	
Overall satisfaction with the quality of school entrepreneurship education (EES5)	3.861	0.957	

**TABLE 5:** The reliability and validation of the study model (*N* = 1223).

	SFL	CR	AVE	α
**Entrepreneurship education curriculum (EEC)**		0.939	0.718	0.939
There are various types of entrepreneurship education curriculum (EEC1)	0.829***			
Entrepreneurship curriculum content closely combined with professional knowledge (EEC2)	0.829***			
Entrepreneurship curriculum content closely combined with cutting-edge trends (EEC3)	0.885***			
Teachers teach in a variety of ways (EEC4)	0.864***			
Teachers have entrepreneurial experience (EEC5)	0.832***			
Teachers have rich experience in entrepreneurship education and teaching (EEC6)	0.844***			
**Entrepreneurship practice (EP)**		0.949	0.755	0.949
There are teachers in and out of campus to guide entrepreneurship practice (EP1)	0.846***			
Entrepreneurial practice is supported by special venture funds (EP2)	0.852***			
The university provides integrated entrepreneurial practice services (EP3)	0.890***			
There are independent student entrepreneurship practice parks (EP4)	0.869***			
There are special off-campus practice bases (EP5)	0.872***			
Entrepreneurial practice programs are highly integrated with professional learning (EP6)	0.885***			
**Satisfaction with entrepreneurship education (EES)**		0.960	0.827	0.959
Enrich entrepreneurial knowledge (EES1)	0.914***			
Cultivate the innovative spirit (EES2)	0.918***			
Improve entrepreneurial skills (EES3)	0.922***			
Inspire entrepreneurial willingness (EES4)	0.898***			
Overall satisfaction with the quality of school entrepreneurship education (EES5)	0.894***			

N = 1223. SFL, standardized factor loading; CR, composite reliability; AVE, average variance extracted. ***p < 0.001.

#### Items of Entrepreneurship Education Curriculum

The dimensions of entrepreneurship education curriculum in this study mainly assessed the curriculum itself and teachers’ teaching. It was composed of six items: types of entrepreneurship education curriculum, combination of entrepreneurship curriculum content with professional knowledge, combination of entrepreneurship curriculum content with cutting-edge trends, teachers’ diverse teaching methods, teachers’ rich experience in entrepreneurship teaching, teachers’ experience in entrepreneurship. The items were scored on a five-point Likert scale. The Cronbach’s α was 0.939.

#### Items of Entrepreneurship Practice

The entrepreneurship practice dimension contained six items, including entrepreneurship practice with guidance from inside and outside the universities, entrepreneurship practice with special entrepreneurship fund support, comprehensive entrepreneurial practice services from universities, entrepreneurship practice with independent entrepreneurship park, and entrepreneurship practice with special off-campus practice base, combination of entrepreneurial practice project and professional learning. The items were scored on a five-point Likert scale. The Cronbach’s α was 0.949.

#### Items of Satisfaction With Entrepreneurship Education

The degree of satisfaction with entrepreneurship education focused on the overall satisfaction of students with the quality of entrepreneurship education. It contained five items: entrepreneurship education that helps enrich entrepreneurial knowledge, cultivates the innovative spirit, improves entrepreneurial skills, inspires entrepreneurial willingness and overall satisfaction with the quality of school entrepreneurship education. The items were recorded on a five-point Likert scale. The Cronbach’s α was 0.959.

## Results and Discussion

### Measurement Model

Confirmatory factors were used to analyze the measurement model of each variable. The factor load range of each item was significant from 0.829 to 0.922, and all *P* values were less than 0.001. The combinatorial reliability (CR) ranged from 0.939 to 0.960, exceeding the benchmark 0.6 which confirms the reliability of combinatorial validity. The average variance extracted (AVE) value of the mean variance variation of latent variables ranged from 0.718 to 0.827, which was within the acceptable threshold (Fornell and Larcker, 1981), indicating ideal convergence validity. Analysis results showed that, *p* < 0.001, χ^2^/*df* = 3.430 < 5, RMSEA = 0.045 < 0.08, RMR = 0.019 < 0.05, GFI = 0.971 > 0.9, TLI = 0.986 > 0.9, AGFI = 0.951 > 0.9, NFI = 0.986 > 0.9, CFI = 0.990 > 0.9, IFI = 0.990 > 0.9, All fitting indexes reached the standard, indicating that the scale had good structural validity (Hair et al., 2010).

### Hypothesis Testing

**FIGURE 2:**
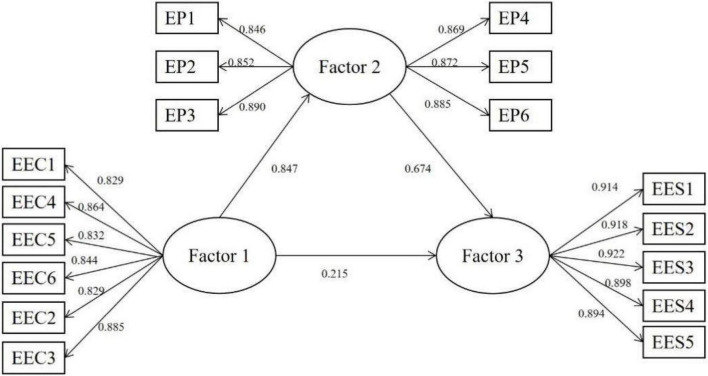
Factor 1, entrepreneurship education curriculum; Factor 2, entrepreneurship practice; Factor 3, entrepreneurship education satisfaction.

The results of the survey were showed in Table 6 and Figure 2. It can be seen that entrepreneurship education curriculum has a significant positive impact on entrepreneurship practice, β = 0.847 > 0, and this path shows a significant level of 0.01 (*Z* = 29.841, *P* = 0.000 < 0.01), So hypothesis 2 is valid. Entrepreneurship practice has a significant positive impact on satisfaction with entrepreneurship education, β = 0.674 > 0, and this path shows a significant level of 0.01 (*Z* = 17.502, *P* = 0.000 < 0.01), So Hypothesis 3 is valid. Entrepreneurship education curriculum has a significant positive impact on satisfaction with entrepreneurship education, β = 0.215 > 0, and this path shows a significant level of 0.01 (*Z* = 5.987, *P* = 0.000 < 0.01). So Hypothesis 1 is valid. Then, we verified the mediating effect of entrepreneurship practice between entrepreneurship education curriculum and satisfaction with entrepreneurship education. Hayes’ PROCESS was used to test the mediating effect of entrepreneurship practice, and the bootstrap (bootstrap = 5000) method was used to test the significance of the mediating effect, β = 0.571 > 0, indicating that entrepreneurship practice has a mediating effect between entrepreneurship education curriculum and satisfaction with entrepreneurship education.

**TABLE 6:** Model regression coefficient table.

Path	SE	CR	*p*	β
EEC → EP	0.027	29.841	***	0.847
EP → EES	0.040	17.502	***	0.674
EEC → EES	0.036	5.987	***	0.215

***p < 0.001.

### Discussion

First, we proved that entrepreneurship education curriculum has a positive impact on agricultural students’ satisfaction with entrepreneurship education. Our results are consistent with previous studies. Chen et al. (2021) proved that teaching methods and teachers’ entrepreneurial attitude are the two biggest variables affecting students’ satisfaction with entrepreneurial education. Kang et al. (2016) showed that the overwhelming majority of students believed that the main reason why entrepreneurship education was unsatisfactory was that the shortage of innovative and high-quality teachers affected the quality of entrepreneurship curriculum. Moreover, other research results also showed that students’ satisfaction with entrepreneurship education is positively correlated with the satisfaction degree of entrepreneurship course construction to students and society (Li, 2017; Pang et al., 2018; Chen et al., 2021), and with the degree to which entrepreneurship curriculum are combined with professional curriculum (Huang and Du, 2020). While, the importance of agricultural development to developing countries has also led to a growing need for students with entrepreneurial knowledge and skills (Hosseini and Pouratashi, 2011). Entrepreneurship education needs to apply different teaching techniques and contents to give full play to its potential (Liñán and Fayolle, 2015; Wiley and Berry, 2015). While previous researchers have shown that entrepreneurship education traditionally focused on imparting personal knowledge, initiatives now focus on action and learning by doing (Rasmussen and Sorheim, 2006). As students’ progress in their entrepreneurship education, they move from knowledge-based, knowledge-oriented curriculum to more applied curriculum (Matlay et al., 2012). Entrepreneurship curriculum are the source of the basic knowledge reserve of entrepreneurship. Therefore, experts with entrepreneurial experience are needed to participate in the curriculum design and teaching (Wu and Chen, 2019). Entrepreneurship education requires teachers to have extensive theoretical knowledge, rich social and work experience. Most universities in China take entrepreneurship curriculum as an extension of management curriculum that taught by management professors and career guidance counselors who lack advanced entrepreneurship academic training or practical entrepreneurship experience (Yu, 2018). Therefore, this needs to be reformed.

Second, entrepreneurship practice has been proved to be a key intermediary variable between entrepreneurship education curriculum and agricultural students’ satisfaction with entrepreneurship education. Existing studies mostly focus on exploring the role of entrepreneurship practice in entrepreneurship education (Pittaway et al., 2009; Pittaway and Edwards, 2012; Wang, 2020a). It is generally accepted that curriculum and knowledge in higher education are particularly constructed through assessment practice (James, 2014). Evaluating the relationship between entrepreneurship practice and their actual entrepreneurial outcome from the perspective of students is the core issue that educators pay attention to. On the one hand, it supports students’ progress; on the other hand, it evaluates students’ performance (Pittaway and Edwards, 2012). Based on the existing literature, we find that there are few studies on the topic of entrepreneurship practice and college students’ satisfaction with entrepreneurship education, let alone agricultural students. There is no denying that entrepreneurial practice is an important indicator affecting students’ satisfaction with entrepreneurship education (Huang et al., 2020b). To be clear, our study further verifies the mediating effect of entrepreneurship practice in the relationship between entrepreneurship education curriculum and students’ satisfaction with entrepreneurship education. What’s more, practical application of entrepreneurship education is the key to promote the implementation and improve the quality of entrepreneurship education (Wang, 2020b). Institutions can offer entrepreneurship practice curriculum to cultivate students’ practical ability and entrepreneurial literacy by focusing on entrepreneurial education with practice-based, incubation-based, and other characteristic methods (Wang, 2020a). For agricultural students, entrepreneurship play a key role in solving agribusiness problems, including the need for water conservation, sustainable packaging, and environmental protection (Higgins et al., 2018). In addition to food production, an essential feature of multifunctional agriculture entrepreneurship is to address how to start agricultural enterprises, especially social enterprises related to agricultural food for responding to rural development and thus promoting socio-economic vitality in rural areas. In the current era of an increasingly rich knowledge economy, entrepreneurship education has undoubtedly become an objective need for the individual development of students. Entrepreneurship curriculum cultivate innovative thinking and consciousness, and entrepreneurial practice can increase practical experience and skills.

## Conclusion

This study investigated a model of agricultural students’ satisfaction with entrepreneurship education that includes entrepreneurship education curriculum and entrepreneurship practice. The research showed that both entrepreneurship education curriculum and entrepreneurship practice can promote agricultural students’ satisfaction with entrepreneurship education. It also showed that entrepreneurship practice plays a mediating role in the relationship between entrepreneurship education curriculum and satisfaction with entrepreneurship education. This means that universities can better promote the implementation of entrepreneurship education from the perspective of entrepreneurship practice and thus improve students’ entrepreneurial knowledge and skills. This study also advanced the understanding of the complex mechanism between entrepreneurship education curriculum and satisfaction with entrepreneurship education.

### Theoretical and Practical Implications

Firstly, students’ satisfaction with entrepreneurship education is not only a factor to be considered in the evaluation of entrepreneurship education curriculum, but also a key index to evaluate the quality of entrepreneurship education (Huang and Du, 2020). The most important and meaningful is that this study pays attention to practical learning, rather than disconnecting entrepreneurship education curriculum and entrepreneurship practice, which provides important enlightenment for teachers to set up entrepreneurship curriculum content and choose teaching methods based on students’ needs. On the one hand, teachers could combine the curriculum content with their own entrepreneurial experience to cultivate the innovative and entrepreneurial thinking of agricultural students (Gao et al., 2021). On the other hand, entrepreneurial teaching methods should be encouraged, such as project teaching method and experiential teaching method, so that students can truly experience how to innovate and start businesses in practice (Chen et al., 2021).

Secondly, this paper expands the theoretical and practical literature on entrepreneurship education for agricultural students. This study focuses on entrepreneurship education for students from other disciplines. Some scholars have conducted previous studies on entrepreneurship education practice and believe that empirical studies on this topic were too few and business schools still occupy a dominant position in entrepreneurship education (Matlay et al., 2012). This study highlights the need to further explore the practice assessment of entrepreneurship education in multidisciplines rather than in business. This study also shows that entrepreneurship education is important in other fields as well. Agriculture itself is a sustainable social entrepreneurship practice (Hudcová et al., 2018) and entrepreneurship has become one (or perhaps the most important) aspect of agriculture in the last decade and will become increasingly important in the near future. Market developments agricultural policy and society have emphasized the need for a higher level of entrepreneurship (Beldman et al., 2014).

Finally, this study provides a structural path for universities to improve agricultural students’ satisfaction with entrepreneurship education through entrepreneurship education curriculum and practices. Existing studies show that institutions of agricultural higher education and universities with agricultural colleges play a very important role in specialized teaching for the agricultural labor market and in training entrepreneurs with agricultural skills and knowledge (Kőmíves et al., 2019). In other words, higher education institutions, especially universities, play an important role in training a large number of agricultural entrepreneurial talents and promoting the development of agricultural industry around the world. While, the role of entrepreneurship education in training agricultural talent can be maximized by exploring the satisfaction of agricultural students to entrepreneurship education and exploring their demand for entrepreneurship education. Meanwhile, the path of combining entrepreneurship education curriculum and practices could improve the professional skills of agricultural students (Student D), increase their entrepreneurial awareness and willingness (Student A), provide entrepreneurial mentors, entrepreneurial bases and alumni entrepreneurship scholarships (Student A), let them learn something useful from the entrepreneurial practice in and out of campus (Student B and C), which could improve agricultural students’ satisfaction with entrepreneurship education to some extent.

### Limitations and Future Research Directions

Although the field is expanding, most studies tend to be fragmented. Early scholars have suggested that most research questions tend to be related to specific curriculum and focus on more direct measures of effectiveness, such as students’ interests and preferences, students’ knowledge acquisition, satisfaction with specific instructors, curriculum content, etc. (Garavan and O’Cinneide, 1994). There are some limitations in this study which can provide enlightenment for future research. First, this study mainly emphasized the two factors of agricultural students’ satisfaction with entrepreneurship education from entrepreneurship education curriculum and entrepreneurial practice. Future research should further explore the relationship between environmental factors and entrepreneurship education satisfaction. In addition, entrepreneurship curriculum designer should consider students’ personal situations and characteristics from different professional fields. Second, this study explored the universality of agricultural students’ satisfaction with entrepreneurship education in China without considering the comparison between cities and countries. Future researchers should use a wider sample and compare various approaches to entrepreneurship teaching in different research fields. However, due to the heterogeneity of enterprise ecosystem in different countries and different cities (Kantis et al., 2020), it will have different impacts on the local entrepreneurial situation. Therefore, the future research direction can be more extensive exploration.

Finally, this study mainly adopted cross-sectional data analysis and failed to grasp the development dynamics of sustainable entrepreneurship among agricultural students. The relationship between agricultural students’ entrepreneurship education and sustainable development needs more in-depth research, for example, what are the mediating variables, and how do they promote sustainable development.

## Data Availability Statement

The original contributions presented in the study are included in the article/supplementary material, further inquiries can be directed to the corresponding authors.

## Ethics Statement

Ethical approval was not required in accordance with laws, regulations, and institutional requirements. Completion of the survey implied the participants’ informed consent.

## Author Contributions

YH: funding acquisition, project administration, supervision, and writing-review. YB: writing-review, questionnaire survey, and editing. LL: conceptualization, methodology, writing-original draft, and editing. DX: supervision, questionnaire survey, and editing. ZX: methodology, questionnaire survey, and editing. GZ: methodology, writing-review, questionnaire survey, and editing. All authors contributed equally to the article.

## Conflict of Interest

The authors declare that the research was conducted in the absence of any commercial or financial relationships that could be construed as a potential conflict of interest.

## Publisher’s Note

All claims expressed in this article are solely those of the authors and do not necessarily represent those of their affiliated organizations, or those of the publisher, the editors and the reviewers. Any product that may be evaluated in this article, or claim that may be made by its manufacturer, is not guaranteed or endorsed by the publisher.
